# Private communication with quantum cascade laser photonic chaos

**DOI:** 10.1038/s41467-021-23527-9

**Published:** 2021-06-07

**Authors:** Olivier Spitz, Andreas Herdt, Jiagui Wu, Grégory Maisons, Mathieu Carras, Chee-Wei Wong, Wolfgang Elsäßer, Frédéric Grillot

**Affiliations:** 1 LTCI, Télécom Paris, Institut Polytechnique de Paris, Palaiseau, France; 2 mirSense, Centre d’intégration NanoInnov, Palaiseau, France; 3 Technische Universität Darmstadt, Darmstadt, Germany; 4 Fang Lu Mesoscopic Optics and Quantum Electronics Laboratory, University of California Los Angeles, Los Angeles, CA USA; 5 College of Electronic and Information Engineering, Southwest University, Chongqing, China; 6 Center for High Technology Materials, University of New-Mexico, Albuquerque, NM USA

**Keywords:** Electrical and electronic engineering, Fibre optics and optical communications, Mid-infrared photonics

## Abstract

Mid-infrared free-space optical communication has a large potential for high speed communication due to its immunity to electromagnetic interference. However, data security against eavesdroppers is among the obstacles for private free-space communication. Here, we show that two uni-directionally coupled quantum cascade lasers operating in the chaotic regime and the synchronization between them allow for the extraction of the information that has been camouflaged in the chaotic emission. This building block represents a key tool to implement a high degree of privacy directly on the physical layer. We realize a proof-of-concept communication at a wavelength of 5.7 μm with a message encryption at a bit rate of 0.5 Mbit/s. Our demonstration of private free-space communication between a transmitter and receiver opens strategies for physical encryption and decryption of a digital message.

## Abstract (web summary)

Free-space communication in the mid-IR domain has many potential applications, but security is still challenging. Here, the authors use chaos synchronization in a QCL-based free-space link as a way to increase privacy of such transmissions.

## Introduction

Free-space communication has experienced a renewed interest in several optical wavelength domains where the atmosphere has high transparency. Experiments in the ultraviolet have recently achieved a transmission at 1 Gbits/s with III-nitride micro-LEDs^1^. In the mid-infrared, several strategies have emerged in order to produce high-bandwidth sources emitting in the transparency window around 4 μm. Communication up to 70 Mbits/s was demonstrated with interband cascade lasers (ICLs)^2^ and transmission at several Gbits/s was achieved with wavelength down-conversion and up-conversion between 1550 nm and 3594 nm, respectively^3^. With quantum cascade lasers (QCLs), transmission at similar high speeds has been reported both at room^4^ and cryogenic temperatures with a free-space television link application^5^.

Despite the substantial investments into development of mid-infrared wavelengths emitters back in the seventies, these systems remained bulky, inefficient, and expensive^6^. The recent accelerated advances in QCLs^7^, as well as progress in mid-infrared detectors^8,9^, enable low-size, low-weight, energy-efficient, and therefore affordable optical systems operating at room temperature in the mid-infrared. The advantage of wavelengths between 3–5 μm and 8–11.5 μm compared to near-infrared wavelengths is that their transmission in the atmosphere remains high even though the presence of fog or haze^10^. Moreover, a transmission between 8 and 11.5 μm implies stealth because the thermal black-body radiation shows a strong background at these wavelengths^11^. Therefore, it would be very difficult to detect transmitted signal reliably in this mid-infrared window and for the moment, only QCLs and CO_2_ lasers can output powerful beams at such wavelengths. QCLs are unipolar semiconductor lasers emitting in the mid-infrared domain^12^ and they are acknowledged for various applications in this wavelength domain^13–17^.

Due to the large data-carrying optical signals propagating free-space, implementation of secure cryptosystems has raised attention. Two options are currently available for those cryptosystems: ghost polarization communication^18^ and quantum key distribution (QKD). The latter has been thoroughly developed during the past 20 years with a focus on visible light and free-space propagation^19^. The current challenge is to realize QKD-based ground-to-satellite communication^20^ and one of the Gordian knots resides in overcoming the background noise that is detrimental during daytime^21^. Propagation conditions are more favorable during night^22^ but this strongly restrains the availability of the transmission link and that is the reason why other wavelength domains are currently investigated. For instance, near-infrared light has drawn attention^23^ because it is immune to parasitic visible-light perturbation and because optical sources are widely available in this wavelength domain known for telecom applications. Though very appealing because it relies on physically uncrackable photon entanglement, QKD for large-scale communication systems is still hindered by the performances of low-speed, low-yield, expensive transmission apparatus^24^. Furthermore, due to the lack of single-photon detectors, QKD technology is not yet available at mid-infrared wavelength that is one of the most relevant transmission domains.

An alternative, nearer-term pathway for free-space, high-bandwidth private optical communication relies on using chaotic waveforms from photonic oscillators^25^. In such a way, the privacy of the transmission mostly results from the fractal dimension and the complexity of the strange attractor where multiscale similarity of the density of trajectories exists^26^. Practically, several methods of encryption were studied with a particular focus on the chaos being the carrier among which the message is hidden. The main idea is to hide a message with a small amplitude within the chaotic signal of the transmitter so that the message does not disturb the larger chaotic fluctuations and remains well concealed from a potential eavesdropper^27^. Indeed, synchronization of two very similar optoelectronic devices, in the following referred to as twins, is robust to small changes in the coupling strength^28^. Consequently, the response-twin-laser, in the following referred to as slave, will still synchronize to the chaos of a chaos-emitting driving twin-laser, in the following referred to as master, even in the presence of a perturbation that is due to message encoding. This mixed signal is then sent to the receiving twin-laser and a detector. As the receiver synchronizes only onto the chaotic fluctuations, subtracting the signal of the receiver from that of the detector allows recovering the concealed message^29^. The proof-of-concept of such configuration in the optical domain was given in 1998^30,31^, followed by the extension to semiconductor lasers under optical self-feedback, which were found to exhibit high-dimensional chaos^32^, leading to high bit-rate private transmission^33,34^ and eventually to a real-field telecommunication system in the metropolitan fiber network of Athens^35^. It is important to underline that this type of communication link is private rather than secure. Indeed, secure would mean that the system is totally unbreakable for anybody whereas private means that it offers a decent way of encryption that is enough to defy a not very motivated eavesdropper. Other methods of communication relying on chaos have been successfully tested through an accurate control over the emitted chaotic fluctuations^36^. The control can be utilized to tune the signal emitted by the chaotic system so that it follows a prescribed symbol sequence, and this allows encoding any desired message from a chaotic oscillator. More recently, optomechanical chaos was also observed in silicon microcavities, opening the way towards synchronous-chaos secure-channel links via chip-scale oscillators^37^. As for free-space communication, only numerical modeling has proven the possibility of a private free-space communication, but for laser diodes in the near-infrared^38^. Our prior works showed that QCLs are, under certain conditions, able to exhibit mid-infrared chaotic oscillations^39,40^.

In this work, we demonstrate a private communication link operating free-space in the mid-infrared window and based on chaos synchronization in QCLs. In our configuration, chaos does not originate from ray dynamics in a laser cavity^41^ but from the temporal dynamics driving the evolution of both photon and carrier densities. The bandwidth of the high-dimensional chaos we generate allows for a transmission rate at 0.5 Mbits/s, and with an error ratio compatible with common telecommunication correction schemes. All in all, the proposed QCL system relying on a message embedded into chaotic waveforms provides superior privacy from hacking.

## Results

### Mid-infrared chaos synchronization and anti-synchronization

**Fig. 1:**
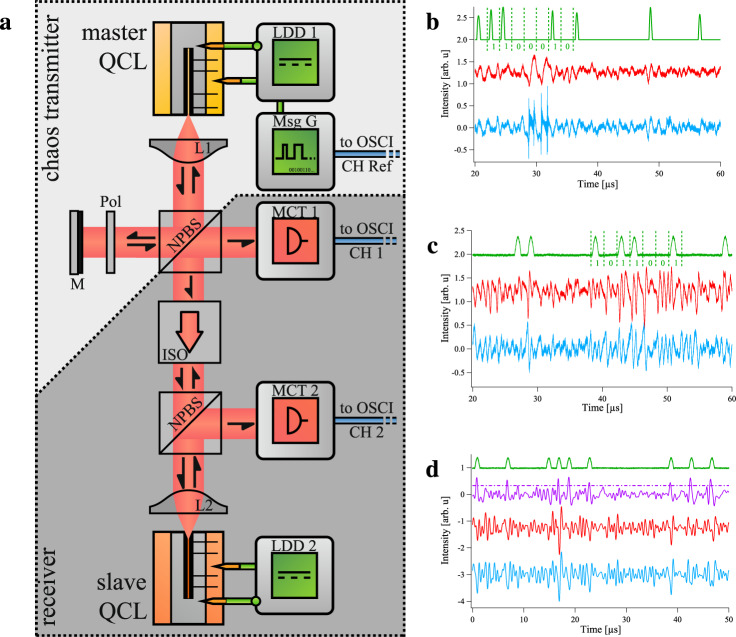
Principle of operation. Left panel: experimental setup showing the transmitter unit (light gray) and receiver unit (dark gray). **a** In the transmitter part, the master laser is driven chaotically by self-optical feedback that is produced by the gold-plated mirror (M). The feedback strength is precisely tuned thanks to a mid-infrared polarizer (pol) with piezoelectric rotation. Thanks to a non-polarizing beam-splitter (NPBS), part of the signal from the master laser is sent into the slave laser through an optical isolator (ISO) to avoid back-coupling. Both the signal from the master QCL and the signal from the slave QCL are retrieved with a MCT detector and then subsequently analyzed. LDD: laser diode driver. Right panel: time series retrieved during the private communication process. **b** Synchronization case. **c** Anti-synchronization case. **d** Filtered time series during message recovery in the anti-synchronization case. The color code is as follows: time trace of the message (green), which is magnified by a factor 30 for a better visualization; time trace of the intensity of the master laser (red) with encoded message hidden within the signal; time trace of the slave laser (blue); time trace of the difference between the red and the blue signal (purple), the dashed line sets the limit between the “0”-bits and the “1”-bits during the analysis of the purple time trace.

After having considered the principles of free-space optics communication, we now define and investigate the progress and our strategy concerning chaos synchronization and message encoding. In the following, the potential eavesdropper will be referred as “Eve” and, by extension, “she” or “her”. The experimental setup that we used for the synchronization of chaos consists out of a distributed-feedback (DFB) master laser driven chaotically by optical self-feedback and a DFB slave laser and is depicted in Fig. 1. In our previous investigations about mid-infrared chaos^40^, we could hardly isolate the narrow chaotic islands of the bifurcation diagram^39^ under continuous operation. This issue mainly came from an imperfect setting of the mid-infrared polarizer in front of the feedback mirror and we solved this problem by placing the polarizer on a piezoelectric rotating mount. Therefore, we are now able to precisely tune the feedback strength and select the one leading to the most appropriate optical chaos. Contrary to some schemes found in laser diodes (see ref. ^42^ for instance), when the master and the slave laser are not coupled, the slave laser is not chaotic because there is no mirror in front of it, so we are in an open-loop configuration. The output of the master is sent towards the slave laser through an optical isolator to avoid back-reflections and the signal of the drive and the response are detected with Mercury–Cadmium–Telluride (MCT) detectors. The signal from the MCT detectors is analyzed with a fast oscilloscope and an RF spectrum analyzer. When the optical frequency of the master and the slave are matching, synchronization occurs and if the master is driven chaotically, the slave will reproduce the same chaotic pattern^43^. Modifying some of the operation conditions, such as the pump current of the master laser or the coupling conditions, can also lead to anti-synchronization^44^, sometimes called reverse synchronization. Though both synchronization and anti-synchronization were achieved in our configuration, we are not able to give a precise set of parameters explaining the switching between synchronization and anti-synchronization. In other semiconductor lasers, these two states can appear for similar conditions and for instance, ref. ^44^ shows an example where the two types of synchronization can be achieved in the same time trace by finely tuning the pump current of the lasers.

In our case, the most relevant results in terms of mid-infrared private transmission were achieved with anti-synchronization and consequently, we will briefly describe a synchronization case before focusing on the anti-synchronization configuration. An example of chaos synchronization with the enciphered message is given in Fig. 1b while an example of chaos anti-synchronization is displayed in Fig. 1c. In the case of anti-synchronization, the filtered time traces can be visualized in Fig. 1d. In the latter, the initial unfiltered message is displayed in green, while the filtered master signal and the filtered slave signal are displayed in red and blue, respectively. The purple time trace corresponds to the difference between the red and the blue signal and a dash-dotted purple line at the constant value 0.33 illustrates the threshold that is used to discriminate 0 and 1 in the purple time trace. One can see that most of the “1”-bits of the purple time trace correspond to the “1”-bits of the green time trace, except for the third “1”-bit of the green time trace. As the master’s signal conveys both the chaotic carrier and the hidden message while the slave’s signal conveys only the chaotic carrier because of the properties of synchronization, the subtraction of the filtered master’s signal by the filtered slave’s signal reproduces a copy of the initial message. The agreement between the original message and its copy will be evaluated hereafter.

As we shall perform communication with two QCLs, the message has to be included in the bias current, with a chaos modulation (CMo) configuration. The voltage-response amplitude of the enciphered bit code is thirty times lower than that of the largest chaotic fluctuations. Indeed, ciphering with a CMo configuration and ensuring that a “1”-bit induces not more than a 10% increase of the average laser intensity are two key requirements for enhanced-privacy chaos transmission^26^. Besides, we observed that privacy is detrimentally affected when increasing the amplitude of the small message signal, and the current configuration with a thirty-time lower response-voltage was the optimized trade-off between privacy and decipherable message after legitimate recovery.

The frequency encoding and modulation pattern were chosen accordingly with the cut-off frequency of the modulation current source (low-pass filter below 2 MHz) and that of the mid-infrared detector (high-pass filter above 1 MHz). This is the reason why an usual non-return-to-zero format could not be selected because many frequencies included in the pattern would be cut either by the current source or by the detector. In order to comply with these constraints, a pulse-up pattern at 1 MHz was chosen because it is composed of very few frequencies outside the frequency range 1–2 MHz. Nevertheless, this was not enough to ensure that the bit is not distorted or creates an undershoot, so a blank always follows a “1”-bit. To sum up, a “1” is encoded with a half-sine and a subsequent blank, while a “0” is encoded with two consecutive blanks. This coding scheme is similar to that which is used in various configurations of high-speed private transmission^45,46^, except that in our configuration, we use a return-to-zero format with 25% duty cycle^47^. It is also worth noting that the code frequency is below the cut-off bandwidth of the chaotic carrier, which is 5 MHz.

### Analysis of transmission with chaos synchronization

**Fig. 2:**
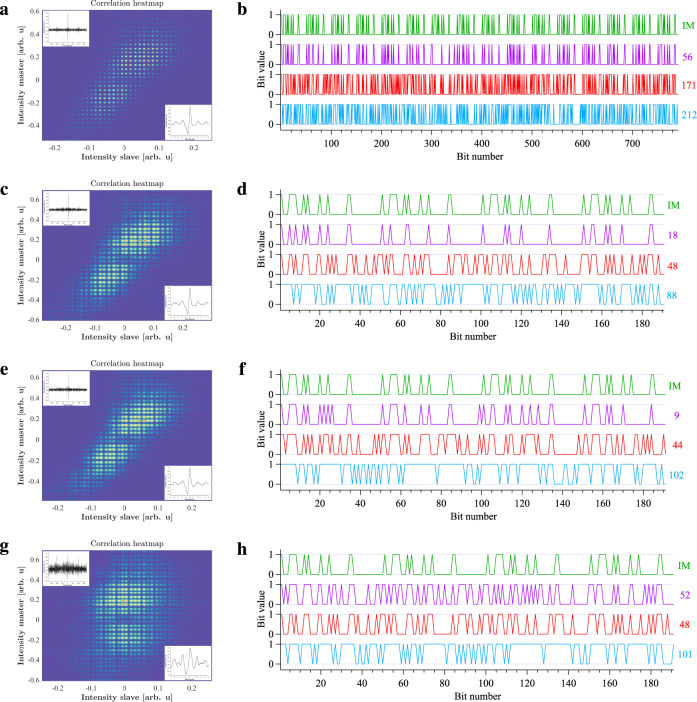
Private communication with chaos synchronization. 2D and 1D (insets) correlation diagrams for the filtered intensity of the slave and the master QCL (left panels). Bit series (right panels) for the initial message (IM in green), the difference (in purple), the master’s signal (in red) and the slave’s signal (in blue). Except for the initial message, the number of errors is written on the right side of the bit series, with the corresponding color. **a**, **b** correspond to the global sequence with 791 bits, **c**, **d** correspond to a portion of the global sequence, between 1 and 191, **e**, **f** correspond to a portion of the global sequence, between 400 and 591, **g**, **h** correspond to a cross-analysis between the slave signal of the second row and the master signal of the third row.

The purpose of the private communication is to transmit a train of 50 bits among which 11 are “1”-bits. The duration of this analysis is 1582 μs, corresponding to 791 bits. Once the message is hidden and the synchronization is achieved, the quality of the transfer is evaluated with correlation diagrams (Fig. 2a), for the filtered time series. The 1D correlation diagram allows retrieving the time lag between the master’s signal and the slave’s signal and is given in insets for two different time scales. This 1D correlation diagram has a large output (close to 1) only for a short range of time, and that confirms the outcome unpredictability of our chaotic signal. The 2D correlation diagrams represent the output power of the slave laser as a function of the output power of the master laser. For a perfect synchronization, the correlation diagram is a straight line with a positive slope and with a Pearson’s correlation coefficient of 1^48^. This line widens and the value of the correlation coefficient decreases down to 0 in case of parameters mismatch between the slave device and the master device^49^. Our synchronization exhibits a correlation coefficient of 0.70. The experimental result of the recovery can be found in Fig. 2b. The recovery chart shows the binary-converted message we want to transmit (in green) and the subsequent bits we retrieved for the master, the slave and the difference (in red, blue and purple, respectively). 212 errors are retrieved when analyzing only the signal of the slave QCL, 171 errors are retrieved when analyzing only the signal of the master QCL and 56 errors are retrieved when analyzing the difference signal between the master and the slave. As our message is made of 11 “1”-bits among 50 bits and our analysis deals with 791 bits, if Eve naively tries to decipher the full transmission only with “0”-bits, she would end with 174 errors. This value confirms that the analysis of the master’s signal alone, even with an appropriate filtering, does not give any details about the transmitted bit sequence. With her bit-error-rate (BER) being 22%, we are also very close to 25% that is the lower limit commonly accepted for a non-decipherable transmission^50^. We further analyzed the previous 791-bit sequence by carrying out the same analysis for portions of the signal. Figure 2c, d describes the correlation analysis and recovery process for bits 1–191, while Fig. 2e–f describes the correlation analysis and the recovery process for bits 401–591. In these two configurations, the correlation plots look similar to those given for the full-length sequence and the subtraction process strongly improves the message recovery. We also decided to plot the same charts when analyzing simultaneously the slave signal from Fig. 2c, d and the master signal from Fig. 2e, f. These results are gathered in Fig. 2g, h and show that the correlation is weak in that configuration and, subsequently, the message is poorly recovered. In this case, the synchronization exhibits a correlation coefficient of 0.20. This information indicates that, even if the message sequence is repeated, this does not imply a repetition of the chaotic pattern used to conceal the message. Thus, this is different from what occurs in the case of a high amplitude periodic modulation leading to synchronized bursts with external optical feedback^40^.

### Analysis of transmission with chaos anti-synchronization

**Fig. 3:**
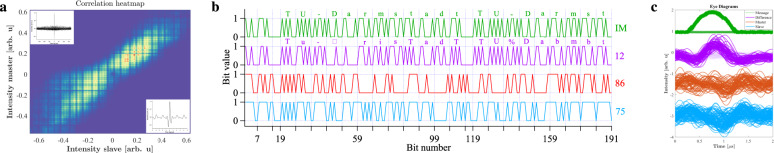
Private communication with chaos anti-synchronization. **a** 2D and 1D (insets) correlation diagrams for the filtered intensity of the slave and the master QCL. The filtered signal of the slave laser was flipped so that the 1D correlation diagram has a maximum value of 1 and the 2D correlation diagram shows a positive correlation (instead of −1 and negative correlation, respectively). **b** Bit series for the initial message (IM in green), the difference (in purple), the master’s signal (in red), and the slave’s signal (in blue). Except for the initial message, the number of errors is written on the right side of the bit series, with the corresponding color. The translation of the difference bit signal is also displayed by comparison with the initial message. The private mid-infrared transmission achieves a BER of 6%, which corresponds to 12 errors out of 191 bits. **c** Experimental eye diagrams for the four time traces displayed in Fig. 1d. From these diagrams, one can see that it is impossible to recover the message only from the signal of the master QCL but it becomes possible for most of the bits from the difference signal. Bits deciphered as “0” are drawn with a light color while bits deciphered as “1” are drawn with a stressed color (particularly visible in the difference eye diagram where the “1” and “0” are well separated).

We now investigate the configuration relying on anti-synchronization to transmit a realistic message which is made of 100 bits with 44 “1”-bits among them. As previously explained, we carry out a correlation analysis for the filtered time series (Fig. 3a), which are also reversed to allow for a better comparison with the synchronization configuration. The 1D correlation diagram allows retrieving the time lag between the master’s signal and the slave’s signal, which is 64 ns in this case. The quality of the correlation is increased in the anti-synchronization case because the correlation coefficient derived from the 2D correlation heatmap has a value of 0.92. The recovery process can be seen illustrated in Fig. 3b and results in 12 errors out of 191 bits. Once again, the master signal gives no clue about the hidden message because the recovery process leads to 86 errors while Eve would obtain 84 errors if she deciphered the sequence only with “0”-bits. This time, her error ratio is 45% and we perform better than in the synchronization case where her BER was 22%, both in the case she would intercept the master signal and apply relevant filtering. This means that we are now in a configuration where Eve performs worse than the limit for a non-decipherable transmission. In order to complement our study, we also display the eye diagram for each component in Fig. 3c. The eye diagram is a tool to evaluate the quality of the transmission and a successful communication is assessed by an open eye^51^. A compromise has to be found between a transmission with a low BER and an impossible recovery for an illegitimate receiver. In this configuration corresponding to a realistic private transmission, the lowest BER we were able to achieve, while ensuring a camouflaged message, is 6%, corresponding to 12 errors out of 191 bits in Fig. 3b. It was possible to achieve lower values of BER but the message started to become obvious among the eye diagram of the filtered master signal and consequently, the privacy of the transmission was threatened. Except for the initial message (green curve), all other eye diagrams are retrieved for the already filtered signals with the best fitting parameters. So this basically means that, even if she knows exactly what is the encrypted message, Eve has absolutely no chance to decipher that message by applying the most relevant filter to the master signal, as it can be visualized from Fig. 3c (red curve) where no indication about the hidden message can be derived.

## Discussion

By taking support from our previous findings about optical chaos, we demonstrated a free-space private communication at mid-infrared wavelength. This achievement was combined through anti-synchronization and message enciphering, with a bit rate of 0.5 Mbits/s and a BER of 6%. It is worth noting that this achievement is also possible with a synchronization scheme, but with degraded performances. Even if the mid-infrared wavelength of this transmission makes it relevant for further applications, improvements must be realized in order to comply with communication standards. Continuous progress in forward error correction (FEC)^52^ allows now envisioning error-free transmission for a BER limit as high as 4%^53^. Consequently, our configuration with a BER of 6% could be used as a real-field system if we can reach a BER of 4%, which is a completely realistic improvement. The price to pay, however, is a reduction of the data rate because such correction requires additional bits during the transmission, which amounts to 27% of the initial message length in the case of a 4% BER limit. Lower values of BER decrease the number of extra correction bits and are thus desirable in our proof-of-concept experiment. It is relevant to note that FEC has a step-like efficiency, so BER below the critical value will be greatly enhanced while BER above the critical value will remain mostly unchanged^50^ and consequently, the privacy of the transmission is not threatened by this technique. Even though CMo gave better results in terms of BER with other semiconductor lasers^54^, it would be interesting to compare this method with chaos masking, for instance using mid-infrared external modulators that already achieve dozens of Mbits/s^55^. Meanwhile, several opportunities can be envisioned in order to enhance the real-field performances of this experimental proof-of-concept. The first one will be to increase the distance between the master and the slave laser because it is currently only 1-m long. If a distance of several kilometers can be achieved, private communication can be well adapted for transmission between ground stations or vehicles and various aircraft. To perform such long-distance communication, it is mandatory to switch from the current 5.7 μm wavelength towards wavelengths that are more suitable for optimal transmission through the atmosphere, for instance 4 μm and 10 μm. While the latter can almost solely be achieved with QCLs, the former can be realized by other semiconductor lasers like ICLs. The most relevant option between these two lasers could be the one that is able to generate chaos with the largest bandwidth. As our private communication system is meant to be mobile, inquiries to find the semiconductor mid-infrared sources with the minimum bulkiness and energy footprint^56^ will also be undertaken.

Further attention will be paid to evaluating the effect of the atmospheric turbulence on the chaos synchronization and this will guide the choice of the most appropriate wavelength. However, a transmission rate of hundreds of Gbits/s can sometimes be required and this does not seem realistic with the aforementioned semiconductor lasers. A relevant option, in this case, would be to transmit only a secret key with the chaotic scheme and then use other means to achieve a very high-speed transmission. As the key can be as small as a few dozens of kbits^57^, the data rate is not an issue because we showed that it was possible to transmit a message at 0.5 Mbits/s in this work. However it is worth noting that, in the case of low-speed private communication, chaos-masking transmission is challenged by QKD. Further investigation can also consider chaos and synchronization in phased-array QCLs from Talbot-based cavity^58^. In such a case, several carrying chaotic emitters for multiple QCL transmitters could be modulated by different message signals to accommodate the larger bandwidth of the carrier. Last but not least, with the emergence of chalcogenide optical fibers^59^, private transmission in the mid-infrared can also be considered in more conventional telecommunication networks, thus encouraging efforts towards higher wavelengths related to the ever-growing demand for communication services.

## Methods

### Synchronization conditions

**Fig. 4:**
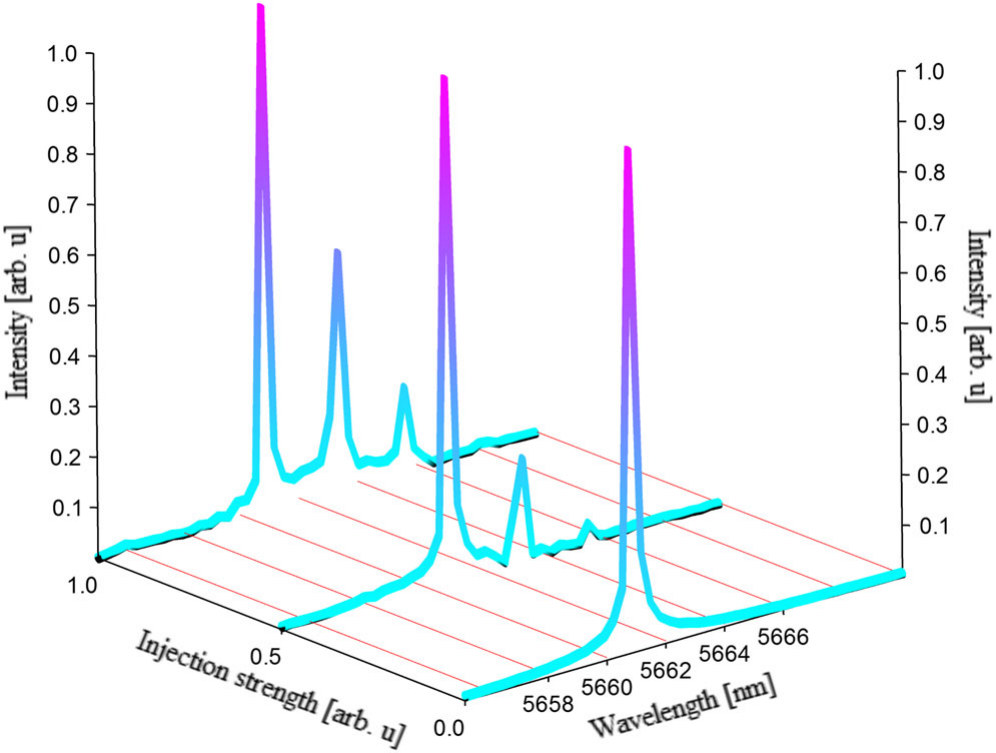
Optical spectrum evolution during the coupling process. Optical spectrum of the slave laser when it is biased at 512.4 mA and for several levels of injection from the master laser. The QCL is monomode under free-running operation and when increasing the injection strength, a second mode is appearing with a subsequently following third mode. The amplitude of these side modes increases with the injection strength while the main mode remains unchanged.

Apart from the chaotic features observed with the RF spectrum analyzer and the digital oscilloscope, it is possible to confirm that the master signal is well injected into the slave by observing the behavior of the optical spectrum with a Fourier transform infrared spectrometer. Indeed, under free-running operation, the slave laser is mono-mode and it turns to be multi-mode when the synchronization is effective, as can be seen in Fig. 4. The broadening of the optical spectrum has been widely observed in semiconductor lasers generating deterministic chaos^60^. However, as already shown with the 2D correlation diagram, the synchronization between the master and the slave is not perfect and consequently, the BER remains high though sufficient for a proof-of-concept experiment. That limitation is due to the differences in the operation conditions for the two lasers in order to match their optical wavelengths, which is a mandatory condition in order to obtain a, yet imperfect, synchronization. In our configuration, the master laser is pumped in continuous mode at 750 mA and the temperature of the device is 251 K. The slave laser is pumped in continuous mode at 510 mA and kept at a temperature of 278 K. These operation conditions were achievable because the two lasers were mounted in separated Newport LDM-4872 sockets, which were further sealed and purged with gas nitrogen. It is worth noting that, apart from the case where the DFB master laser and the DFB slave laser have the same optical wavelength, it is also possible to achieve synchronization (or anti-synchronization) when the main optical frequency of the master laser is equal to that of one of the suppressed side-modes of the slave laser. Practically, this means that changing the bias current of the slave laser allows scanning the optical frequency and achieving synchronization around the aforementioned side-modes. Such current values are gathered in Table 1 and a deviation as small as 1 mA from these values hinders the synchronization because the slave QCL is no longer injection-locked to the master QCL. Indeed, the synchronization can only be possible if the master and the slave laser are in the injection-locking range. Although these multiple current values could ease the task for Eve, it is important to remember that QCLs are currently more difficult and more expensive to grow than conventional laser diodes or VCSELs. As this work is a proof-of-concept experiment, we did not analyze all the parameters which could lead to a degraded correlation between the master and the slave. In other semiconductor lasers, the key parameters to ensure a reliable synchronization are the photon lifetime, the carrier lifetime, the gain and the linewidth enhancement factor^61,62^. In our experiment, we also did not test the influence of the distance between the two lasers, which was fixed at one meter. However, we varied the injection strength and this operation did not cause any obvious degradation of performance even with an injected optical power of a few mW at the slave level. Considering that our master laser can generate up to 240 mW of optical power, this means that the beam can undergo an attenuation of roughly 20 dB, which is equivalent to a few kilometers transmission at mid-infrared wavelength. However, this does not take into account the multiple distortion and beam expansion during the transmission path. Being able to perform the synchronization even with reduced injected power in the case of QCLs is in good agreement with laser diodes experiments exhibiting a wide coupling window^63^.

**Table 1:** Bias currents of the slave QCL leading to synchronization when the bias current of the master QCL is fixed at 750 mA. The temperature of the master laser is fixed at 251 K while the temperature of the slave laser is fixed at 278 K.

Bias current (mA)	370	400	429	457	484	510	538	562	586	608

### Optical sources

Technology of quantum devices developed by mirSense was used to fabricate DFB QCLs under study. Both their intensive (e.g., cavity length, facet coatings, etc.) and extensive parameters (e.g., carrier lifetime, gain, etc.) were matched to the best extent. The lasers emit single-mode radiation around 5.7 μm with 30 dB side-mode suppression and exhibit a wavelength shift when varying the temperature or current of operation. The order of magnitude of wavelength shift with continuous bias is 10 nm A^−1^. The two QCLs are however different in terms of output power since the master laser is able to output up to 240 mW at 251 K, while the slave laser generates up to 15 mW at 278 K. The discrepancy in temperatures is explained by the strong requirement of wavelength matching between the master and the slave. The devices under study were grown by molecular beam epitaxy on an InP cladding and incorporates 30 periods of AlInAs/GaInAs layers^64^. The upper InP cladding is n-doped at a value of 10^17^ cm^−3^ in order to get electrical injection but without introducing any plasmonic effects. The design for the DFB laser uses index coupling and metal grating^65^. This enables single-mode emission using a top metal grating with a coupling efficiency of *κ* ≈ 4 cm^−1^. The slave and the master QCL come from the same batch and have the same internal cavity length, which means that their microscopic parameters such as carrier lifetime, gain and photon lifetime are almost identical. These parameters are important to ensure optimal synchronization. These lasers emit a continuous wave thanks to a standard double trench configuration without Iron doped Indium Phosphide regrowth for thermal heat sinking optimization. The back-facet of these QCLs is composed of a high-reflectivity coating and this means that the lasers only output mid-infrared light at one side that is left as-cleaved. This emitting facet has a transmission of 30% that is suitable for feedback experiments.

### Recovery process

In order to achieve proper enciphering, the message concealed within the chaotic carrier has a limited bandwidth and amplitude compared to the chaotic signal^66^. Consequently, the recovery process begins with the filtering of the master (containing the message) and of the slave signal. Table 2 assembles the characteristics of our passband filter. We do not use a lowpass filter in our work because we want to get rid of both the noise at high frequency and of the low-frequency contribution because the MCT detectors we used are not able to retrieve frequency components below 0.25 MHz. Indeed, the tabulated 3 dB bandwidth value is 1 MHz but we could observe signals down to a few hundreds of kHz. After filtering, the reconstruction of the message *M*(*t*) is realized by computationally evaluating the difference intensity of the chaos transmitter *I*_ct_ = ∣*E*_master_(*t*) + *M*(*t*)∣^2^ and the intensity of the chaos-synchronized slave *I*_slave_ = ∣*E*_slave_(*t*)∣^2^ ≃ ∣*E*_master_(*t*)∣^2^ resulting in ${I}_{\mathrm{ct}}-{I}_{\mathrm{slave}}=2\Re \left({E}_{\text{master}}\cdot M(t)\right)+| M(t){| }^{2}$.

**Table 2:** Characteristics of the passband filter applied to the master’s signal and the slave’s signal before subtraction.

Parameter	Value
Passband low frequency	0.250 MHz
Passband high frequency	2.000 MHz
Passband ripple	0.1 dB
Stopband attenuation	60 dB
Stopband low frequency	0.137 MHz
Stopband high frequency	2.113 MHz

## Supplementary information

Supplementary Information

## Data Availability

All relevant data are available from the authors upon reasonable request.

## Code availability

Matlab code is also available from the authors upon reasonable request.
