# Supplementary material for: Private communication with quantum cascade laser photonic chaos
Source: Nat Commun. 2021 Jun 7;12:3327. doi: 10.1038/s41467-021-23527-9 (PMC8184773; doi:10.1038/s41467-021-23527-9)
Supplement: Supplementary file 1 — Supplementary Information [file 41467_2021_23527_MOESM1_ESM.pdf]

Supplementary information: Private communication with quantum cascade laser photonic chaos

Olivier Spitz[1,2,*], Andreas Herdt[3], Jiagui Wu[4,5], Grégory Maisons[2], Mathieu Carras[2], Chee-Wei Wong[4], Wolfgang Elsäßer[3], and Frédéric Grillot[1,6]

[1]LTCI, Télécom Paris, Institut Polytechnique de Paris, 19 Place Marguerite Perey, Palaiseau, 91120, France
[2]mirSense, Centre d'intégration NanoInnov, 8 avenue de la Vauve, Palaiseau, 91120, France
[3]Technische Universität Darmstadt, Schlossgartenstraße 7, D-64289 Darmstadt, Germany
[4]Fang Lu Mesoscopic Optics and Quantum Electronics Laboratory, University of California Los Angeles, Los Angeles, CA 90095, USA
[5]College of Electronic and Information Engineering, Southwest University, Chongqing, 400715, China
[6]Center for High Technology Materials, University of New-Mexico, 1313 Goddard SE, Albuquerque, NM 87106, USA
[*]olivier.spitz@telecom-paris.fr

Supplementary discussion

Based on Shannon's theory of secrecy,[1] confusion and diffusion are two properties used to make ciphers robust against statistical analysis. Cracking a high order QCL chaotic waveform is extremely difficult due to both practical, technological, physical and mathematical limitations associated with the degree of complexity of a chaotic behavior.[2] Moreover, we would like to emphasize that our system is not intended to be primarily fiber-based, so the expected mobility of the transmitter and the receiver poses another challenge to Eve. The practical limitations to hacking the system are:
1. The QCL used by Eve must be the same as ours at the microscopic level, with the same physical parameter settings (which can be changed rapidly as part of the encryption protocol). Thus, using another QCL or other types of lasers combined with other detectors is very unlikely to succeed in recreating the initial QCL system in order to hack a transmission or in order to inject an intelligible signal into the data stream for jamming purposes.[3]
2. A transmission between 8-11.5 micron wavelengths along with chaotic waveforms would further provide stealth/masking for the signal. This is due to the random thermal blackbody background radiation,[4] hence reducing the probability of adversaries intercepting the transmission signal.
3. Quantum computers, neural networks or other computing architectures, which can potentially crack standard mathematical encryption via solving elliptical, parabolic or hyperbolic equations, are not necessarily capable of quickly cracking non-quantum dynamical chaos. This means that, in our configuration, the time to decipher the message with mathematical tools is several orders of magnitude larger than the total duration of the transmission itself: Eve may only translate the message long after its validity expired.
So, there are extreme technological and physical barriers[5] in order to attempt to predict and crack an optical chaotic system. In addition to the technological barriers, there are also mathematical barriers to cracking the QCL chaotic waveform, which are:
4. QCL chaotic waveform sources exhibit strong nonlinear dynamical topology structures and fractional dimensions of oscillation (i.e. hyperchaos), which is exactly what is needed for private optical communication links.[6] This property may strongly be reinforced by using an array of coupled QCLs.
5. Nonlinear chaotic systems with only one positive Lyapunov exponent are not robust enough to cracking (i.e. the chaotic signal can be destroyed by further arbitrarily small perturbations of the system). In other words, non-robust chaotic system dynamics enable just one variable to enslave a set of differential equations and thus, these systems will have only one positive Lyapunov exponent.[7]

Accordingly, due to these specific and intrinsic attributes of QCL chaotic systems, one insists that the message embedded in a chaotic waveform offers a decent level of privacy from hacking. This accounts for Eve actively hacking chaotic waveforms, either considering current or great advances in future technological capabilities relying on machine learning and reservoir computing, which are currently used for non-ideal chaos prediction.[8] Our current achievements make the described private-link configuration relevant in the case of operational short-message transmission, for instance between two tanks in the

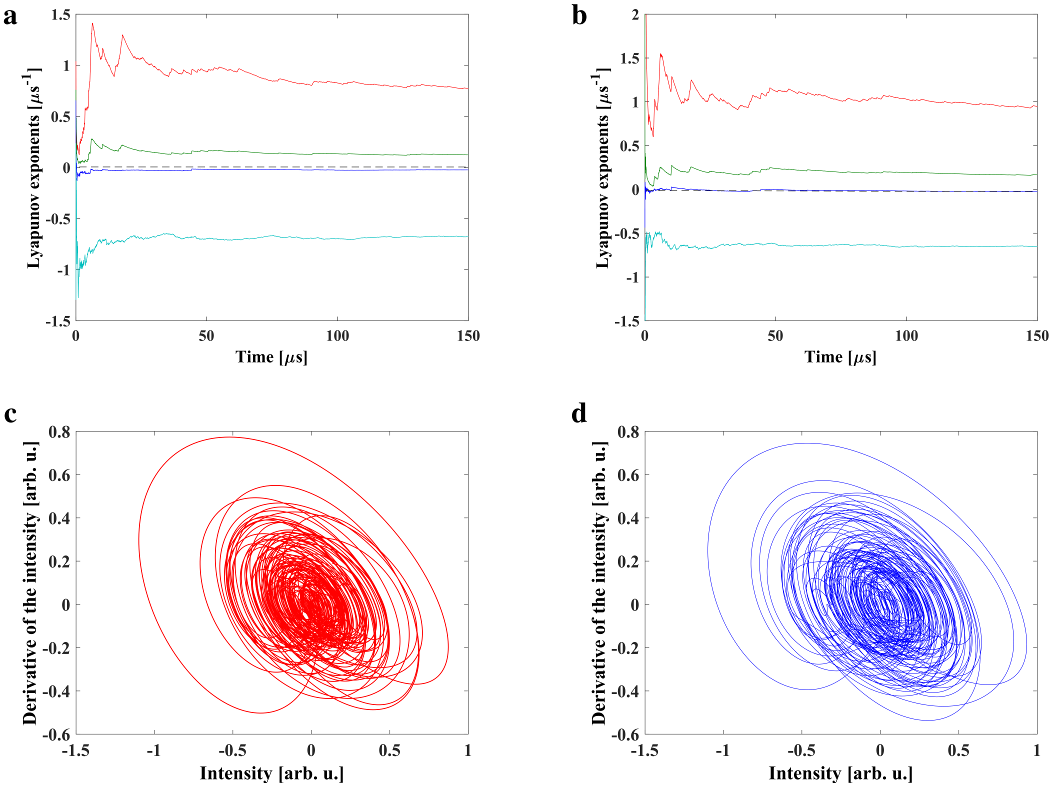

Supplementary figure 1. Comparison of the chaos properties. a-b System dynamics analysis through Lyapunov exponents (LEs). The four largest LEs are displayed and the chaotic behavior is confirmed by the two LEs converging towards values above zero. **c-d** Reconstructed orbital phase portraits for the filtered chaotic master laser and the filtered chaotic slave laser. The two phase portraits look similar, thus confirming the chaos synchronization between the two QCLs. The left panel is associated to the master's signal while the right panel is associated to the slave's signal.

battlefield. With our method, it is very difficult for an eavesdropper to even know that a transmission between two vehicles occurs because the wavelength of the transmission relates to the wavelength of the background environment and because the laser beam is directional. Another application is short-range communication between two rovers on Mars. These rovers have a very low speed, around 1 km/h, but they need to exchange information and free-space optics transmission raised attention. The two advantages of our private transmission scheme are a directional line-of-sight communication immune to the few nearby potential eavesdroppers (for instance other rovers or satellites) and a wavelength of operation very resistant to degraded conditions such as sandstorms.

Supplementary methods

At first, a chaotic waveform may be not distinguishable from a stochastic noisy time trace and consequently, it is mandatory to have tools such as the Lyapunov exponents (LEs) in order to characterize the observed dynamics. The basic idea of the Lyapunov exponent λ is to measure the rate at which two originally nearby trajectories diverge in time, because two close trajectories are supposed to exponentially diverge in the case of chaos.[9] As the direction of the maximum divergence or convergence locally changes on the attractor, the motion must be monitored at each point along the trajectory. On the one hand, at least one of the LEs must be positive to comply with the sensitive dependence on the initial conditions, which is an endemic property of chaotic systems. On the other hand, constant and periodic signals are linked to zero or negative LEs because this means that nearby points converge. Several methods for the calculation of the LEs from experimental time series have been developed .[10–14] In general, the number of LEs that can be retrieved corresponds to the number of system variables and only the largest exponents are derived in order to determine if the system is chaotic. We extract the largest LEs from our 1 million data points filtered time series, recorded with a sampling rate of 2.5 GSamples/s, by using the technique exposed in Ref. 15. Besides, the noise-reduced data are also relevant to reconstruct the phase space trajectories[16] because this confirms the similarities between the chaos from the master QCL and that from the slave QCL, though the latter is starting from an arbitrary condition.[17] The experimental phase diagrams can be found in Fig. 1 **c** and **d**. The analysis of the master signal shows

ASCII	binary	mistaken ASCII	mistaken binary
T	01010100		
U	01010101	u	01110101
-	00101101	%	00100101
D	01000100	□	00000100
a	01100001		00100000
r	01110010	b	01100010
m	01101101	i	01101001
s	01110011	b	01100010
t	01110100	T	01010100
a	01100001		
d	01100100		
t	01110100	T	01010100

Supplementary table 1. Binary code used to transmit the message ”TU-Darmstadt” and associated errors that can be found in the recovery process.

that the four largest LEs converge towards $\lambda_1 = 0.76\mu s^{-1}$, $\lambda_2 = 0.12\mu s^{-1}$, $\lambda_3 = -0.02\mu s^{-1}$ and $\lambda_4 = -0.67\mu s^{-1}$, and the analysis of the slave signal shows that the four largest LEs converge towards $\lambda_1 = 0.94\mu s^{-1}$, $\lambda_2 = 0.16\mu s^{-1}$, $\lambda_3 = -0.02\mu s^{-1}$ and $\lambda_4 = -0.65\mu s^{-1}$, as visualized in Fig. 1 **a** and **b**, respectively. Consequently, the signal generated by the master QCL is considered as hyperchaos[18] because two LEs are positive and this confirms the potential of our setup for private transmission, contrary to systems producing chaotic outputs with only one positive LE.[19]

A key parameter is the technique used to encipher the message within the chaotic carrier. Two methods relying on the concealment of a message within a chaotic carrier have been explored in the literature and they have a tremendous influence on the lowest achievable BER.[20] The first method is called chaos masking (CMa).[21] It consists in embedding a small message $m(t)$ into a chaotic carrier $x(t)$ at the drive level and then, sending the signal $x(t)+m(t)$ to the response. Only the chaotic signal $x(t)$ is reproduced in the response system if the amplitude of the message is small enough. Then, the message $m(t)$ is deciphered by subtracting the response output $x(t)$ from the transmission signal $x(t)+m(t)$. The success of the message private concealing into a chaotic carrier and of the decoding of the message relies on a message amplitude sufficiently small compared with the averaged chaotic carrier signal. Usually, the fraction is less than 1% of the average chaotic power.

The second method is called chaos modulation (CMo).[22] In this method, a message is inserted within a chaotic carrier in the nonlinear oscillator and the two signals conform a new chaotic state different from the original one. In CMo, a delayed feedback system is usually used as a chaotic generator. This new signal together with the message is sent to the response. Since the drive and the response are the same nonlinear systems, the chaotic oscillation is exactly reproduced in the response system thanks to chaos synchronization. By subtracting the synchronized chaotic signal from the transmitted signal, it is possible to decode the message. Sometimes, the message is decoded by dividing the transmitted signal by the synchronized chaotic signal in the response. Contrary to the previous method, CMo has no restriction on the magnitude of the message, since both the chaotic carrier and the message conform new chaotic states in the nonlinear systems. However, the degree of security for data transmission becomes worse when the signal level of a message is large. Therefore, the amplitude of a message in CMo should also be small.

Several possibilities exist in order to convert alphanumeric characters into a code that is compatible with optical transmission. At least 7 bits are required to encode any ASCII character in binary. In practice, an 8th bit is added and used as a parity bit to detect transmission errors and this standard is called UTF-8. Table 1 contains the UTF-8 code we used to transfer our message. Because transmission is not flawless, the transmission errors that we possibly encountered are also gathered in that table.

Supplementary References

1. Shannon, C. E. Communication theory of secrecy systems. The Bell system technical journal 28, 656–715 (1949).

2. Anishchenko, V. Vadivasova, T. Okrokvertskhov, G. & Strelkova, G. Correlation analysis of dynamical chaos. Physica A: Statistical Mechanics and its Applications 325, 199–212 (2003).

3. Rizomiliotis, P. Bogris, A. & Syvridis, D. Message origin authentication and integrity protection in chaos-based optical communication. IEEE Journal of Quantum Electronics 46, 377–383 (2010).

4. Majumdar, A.K. & Ricklin J.C. Free-space laser communications: principles and advances, vol. 2 (Springer Science & Business Media, 2010).

5. Razeghi M. Technology of quantum devices (Springer, 2010).

6. Goedgebuer, J.-P. Levy, P. Larger, L. Chen, C.-C. & Rhodes, W.T. Optical communication with synchronized hyperchaos generated electrooptically. IEEE Journal of Quantum Electronics 38, 1178 (2002).

7. Wernecke, H. Sándor, B. & Gros, C. Chaos in time delay systems, an educational review. Physics Reports 824, 1–40 (2019).

8. Pathak, J. Lu, Z. Hunt, B.R. Girvan, M. & Ott, E. Using machine learning to replicate chaotic attractors and calculate Lyapunov exponents from data. Chaos: An Interdisciplinary Journal of Nonlinear Science 27, 121102 (2017).

9. Ohtsubo, J. Semiconductor lasers: stability, instability and chaos, vol. 111 (Springer, 2012).

10. Sano, M. & Sawada, Y. Measurement of the Lyapunov spectrum from a chaotic time series. Physical Review Letters 55, 1082 (1985).

11. Eckmann, J.-P. Kamphorst, S.O. and Ruelle, D. Ciliberto, S. Liapunov exponents from time series. Physical Review A 34, 4971 (1986).

12. Rosenstein, M.T. Collins, J.J. & De Luca, C.J. A practical method for calculating largest Lyapunov exponents from small data sets. Physica D: Nonlinear Phenomena 65, 117 (1993).

13. Wolf, A. Swift, J.B. Swinney, H.L. Vastano, J.A. Determining Lyapunov exponents from a time series. Physica D: Nonlinear Phenomena 16, 285 (1985).

14. Bryant, P. Brown, R. & Abarbanel, H.D.I. Lyapunov exponents from observed time series. Physical Review Letters 65, 1523 (1990).

15. Brown, R. Bryant, P. & Abarbanel, H.D.I. Computing the Lyapunov spectrum of a dynamical system from an observed time series. Physical Review A 43, 2787 (1991).

16. Schreiber, T. Determination of the noise level of chaotic time series. Physical Review E 48, R13 (1993).

17. Pecora, L.M. & Carroll, T.L. Synchronization in chaotic systems. Physical Review Letters 64, 821 (1990).

18. Qi, G. van Wyk, M.A. van Wyk, B.J. & Chen, G. On a new hyperchaotic system. Physics Letters A 372, 124 (2008).

19. Perez, G. & Cerdeira, H.A. Extracting messages masked by chaos. Physical Review Letters 74, 1970 (1995).

20. Liu, J.-M. Chen, H.-F. & Tang, S. Synchronized chaotic optical communications at high bit rates. IEEE Journal of Quantum Electronics 38, 1184 (2002).

21. Morgül, Ö. & Feki, M. A chaotic masking scheme by using synchronized chaotic systems. Physics Letters A 251, 169 (1999).

22. Halle, K.S. Wu, C.W. Itoh, M. & Chua, L.O. Spread spectrum communication through modulation of chaos. International Journal of Bifurcation and Chaos 3, 469 (1993).
